# Gastric Trichobezoar: A Case Report of a Young Adult From a Secondary Hospital

**DOI:** 10.1002/ccr3.71798

**Published:** 2026-01-02

**Authors:** Mohaned Altijani Abdalgadir Hamdnaalla, Mohammed Ali Mohammed Ali, Eltayeb Sabeil Abdalla Idris, Maisara Fathi Ahmed Salih, Heyam Abdelazim Abdelrahim Ali, Rame Taha Zeiada Taha, Loai Osman Elrofaie Osman

**Affiliations:** ^1^ Internal Medicine Dongola Specialized Hospital Dongola Sudan; ^2^ General Surgery Dongola Specialized Hospital Dongola Sudan

**Keywords:** gastroenterology/hepatology, general medicine, mental health, psychiatry, surgery

## Abstract

**Key Clinical Message**

Trichobezoar is the pathological formation of a hair mass within the gastrointestinal tract, usually correlated with psychiatric conditions like trichotillomania and trichophagia. Intestinal obstruction represents a significant potential complication. Trichobezoar should be included in the differential diagnosis for young females exhibiting nonspecific gastrointestinal symptoms accompanied by abnormal psychiatric behavior.

## Graphical abstract

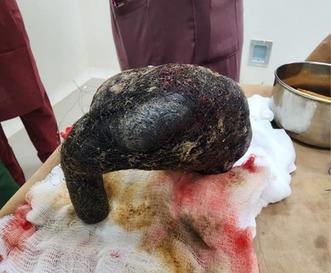

## 1 Introduction

A bezoar is defined as an abnormal aggregation of indigestible materials formed in the gastrointestinal tract. The word “trich” is originally a Greek word that means hair. A trichobezoar specifically denotes a hair mass formed in the gastrointestinal tract. This condition is commonly associated with trichotillomania and trichophagia [1].

Trichotillomania is a psychological disorder characterized by an involuntary and irresistible urge to pull out one's hair. Patients usually extract hair from different sites, such as the scalp, which is the most common area, the eyebrows, and any other area with hair growth. This behavior is notably difficult to control [2].

On the other hand, trichophagia refers to the behavior of eating hair. A psychiatric condition that can precipitate serious complications, including intestinal obstruction. This obstruction results from the formation of a hairball within the gastrointestinal tract, which accumulates and prevents the normal peristaltic movements. Trichophagia is commonly seen in females and is considered the primary etiological factor of trichobezoar [3].

This report describes a rare presentation of trichobezoar in a young adult patient at Dongola Specialized Hospital in Northern State, Sudan.

## 2 Case History/Examination

A 21‐year‐old female has been having compulsive pulling and eating hair for the past 4 years. Throughout this period, she reported no significant complaints except for intermittent epigastric pain, described as colicky, non‐radiating, without specific relieving or aggravating factors. Recently, the patient presented complaining of dyspepsia and vomiting for 1 month. Her oral intake had decreased significantly, and her family observed notable weight loss. She has no significant past medical history, and no additional psychological symptoms were reported by her family. The patient is not currently on any medications and has no known drug allergies. There is no family history of similar conditions or other psychological problems. Physical examination revealed a mildly tender, firm upper abdominal mass that is not attached to the skin over it and has no associated cutaneous changes. No previous surgical scars, no other masses were detected, and no dilated veins.

## 3 Differential Diagnosis, Investigations and Treatment

**FIGURE 1:**
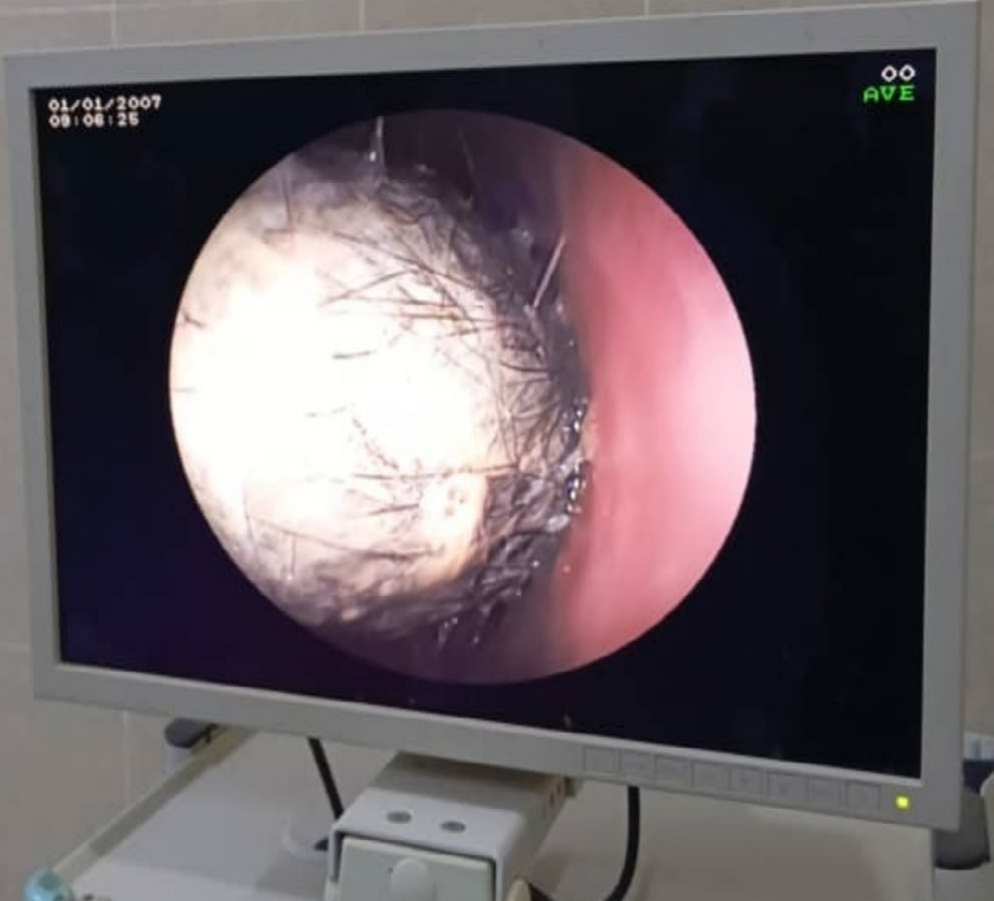
An upper gastrointestinal endoscopy shows a hairy mass inside the gastric cavity.

**FIGURE 2:**
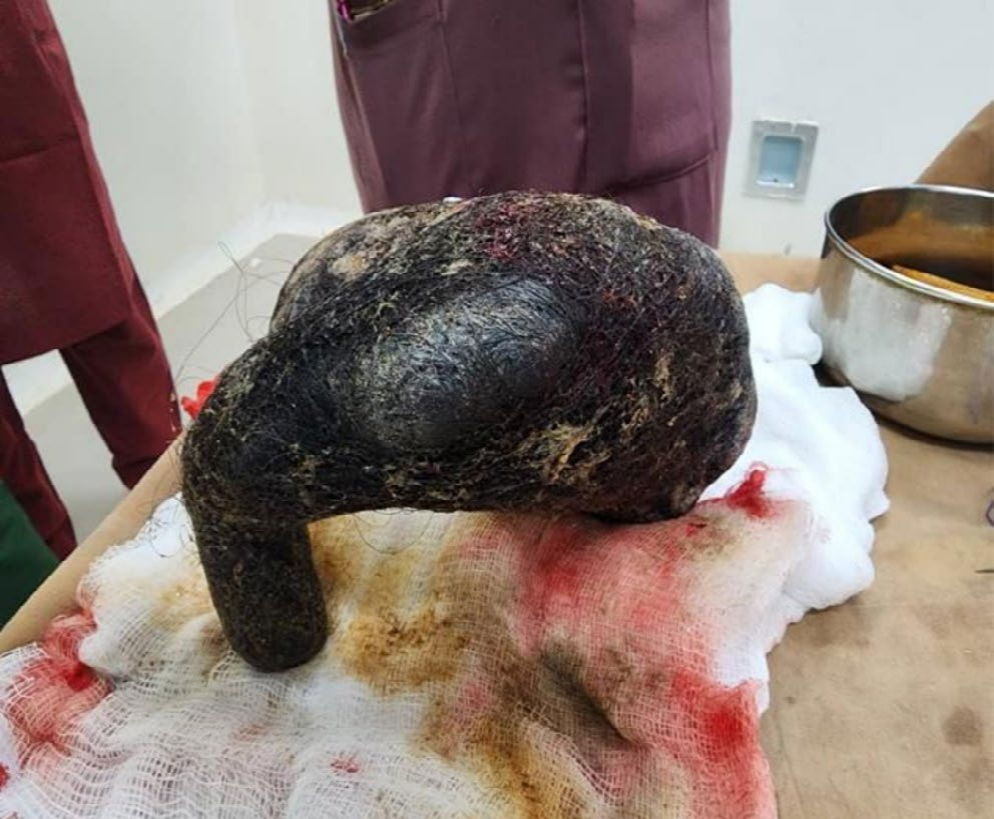
A huge trichobezoar after surgical removal.

Laboratory investigations revealed a high platelet count of 639 × 10^3^ u/L; otherwise, complete blood count, urine analysis, and other routine investigations were normal. Abdominal ultrasound revealed a left upper abdominal mass with peripheral gas shadows. Computerized Tomography of the abdomen was performed and demonstrated a huge heterogeneous mass in the gastric cavity, raising suspicion of gastric bezoar. Upper gastrointestinal endoscopy was performed and revealed a huge hairy foreign body completely occluding the gastric cavity, impending passage (Figure 1). The patient underwent laparotomy via midline incision with anterior gastrotomy, followed by successful surgical removal of a large hairball without intraoperative complications (Figure 2). Exploration of the remainder of the gastrointestinal parts revealed no other bezoar tissues; the abdominal wall was closed using vertical mattress suture.

## 4 Outcome and Follow Up

The patient recovered smoothly thereafter and was referred to a psychiatric specialist.

## 5 Discussion

A trichobezoar is a mass composed usually of undigested hair that accumulates in the stomach. This rare condition is primarily associated with psychiatric problems such as trichotillomania and trichophagia. Trichotillomania refers to the compulsive and abnormal behavior of hair pulling, while trichophagia denotes the eating of that pulled hair. The black color of trichobezoar refers to the acid of the stomach, which denatures the hair's protein. It is estimated that approximately only 1% of individuals with trichophagia can develop trichobezoar [4].

Other types of bezoars include Phytobezoars, which are an accumulation of vegetable fibers. Pharmacobezoars are an accumulation of undissolved medications. Lactobezoars consist of undigested milk protein and mucus tissues. Foreign body bezoars, composed of various foreign materials like paper, plastic, foam, and so on [5].

Studies indicate that most patients with trichobezoar are predominantly females, typically within the range of 13–20 years, with an average age of onset of 11 years. Approximately 80%–90% of trichobezoar are confined to the stomach; sometimes they can be found in the small intestine and the colon. This Phenomenon may be related to Rapunzel Syndrome, where a gastric trichobezoar grows a tail‐like extension through the pyloric sphincter. Small hair strands become trapped in the stomach, but as the mass grows larger, it extends into the duodenum, jejunum, ileum, and sometimes even the colon, potentially causing intestinal obstruction [4].

In extremely rare conditions, trichobezoars may be found in a paediatric population. A case series reported by Murad Habib described six paediatric patients whose ages ranged from 6 to 12 years; all patients presented with non‐specific symptoms similar to those observed in our case and were managed surgically via explorative laparotomy [6].

The clinical picture of trichobezoar ranges from asymptomatic presentations to serious complications. Common symptoms include abdominal pain, nausea, vomiting, anorexia, constipation, and weight loss [7]. As the mass enlarges, it may become palpable upon physical examination. At some point, it may lead to pylorus or intestinal occlusion, which leads to intestinal obstruction. Diagnosis of trichobezoar may be challenging due to the non‐specific nature of symptoms; therefore, imaging modalities such as ultrasound, conventional upper gastrointestinal radiography, computerized tomography (CT), magnetic resonance imaging (MRI), and upper gastrointestinal endoscopy are essential for accurate identification. Most cases are effectively treated surgically via exploratory laparotomy; other therapeutic modalities may have limited efficacy [8].

Trichobezoar remains a rare condition. Less than 500 cases of trichobezoar were reported [8]. The first documented case in Sudan was reported in 2012 [9]. In our case, a young girl presented with non‐specific symptoms, which progressed to severe symptoms accompanied by poor oral intake. Physical examination revealed a palpable abdominal mass, prompting imaging studies such as ultrasound and computerized tomography, which increased the suspicion of a foreign material. The diagnosis was confirmed via upper gastrointestinal endoscopy. We managed the patient surgically with an exploratory laparotomy. These clinical findings are similar to a study from Northern Sudan by Dr. Mohamed Osman Abdelaziz [8], where a 16‐year‐old girl presented with comparable symptoms; upper gastrointestinal endoscopy revealed a hairy mass. Laparotomy was a successful way of management. Published cases from Sudan are summarized (Table 1). Similarly, a study by Anoop Dixit [10] documented a similar presentation managed surgically.

**TABLE 1:** Cases of trichobezoar in Sudan.

Title	Authors	Date of publication	Age of the patient	Management
Trichobezoar‐a‐case‐report‐from‐the‐northern‐state‐sudan	Mohamed Osman Abdelaziz	June 2021	16 years	Surgical removal by laparotomy
Rapunzel syndrome: a case report from al‐dabba hospital, northern state, sudan [10]	Hassan M. E. Eltigani, Sara O. A. Ahmed	April 2024	16 years	Surgical removal by laparotomy

*Note:* Table 1 Those cases were published successfully by the authors.

Multiple treatment options exist for trichobezoar removal, including endoscopic, laparoscopic, and open surgical (laparotomy) removal. Surgical management has demonstrated a successful rate of approximately 99%. Non‐surgical methods such as enzymatic therapy using papain, cellulase, or acetylcysteine have shown high failure rates and are therefore not recommended [10].

## 6 Conclusion

Trichobezoar is indeed an uncommon condition; thus, it is important to include it as one of the differential diagnoses when evaluating a young female presenting with nonspecific gastrointestinal symptoms, particularly if there is any history of abnormal psychiatric behavior. Among the various treatment options, surgical approaches such as laparotomy should be considered in the management of trichobezoar, as they show a high success rate among other modalities.

## Author Contributions

**Mohaned Altijani Abdalgadir Hamdnaalla:** conceptualization, data curation, investigation, project administration, resources, software, supervision, validation, visualization, writing – original draft, writing – review and editing. **Mohammed Ali Mohammed Ali:** data curation, investigation, project administration, resources, validation, visualization, writing – review and editing. **Eltayeb Sabeil Abdalla Idris:** data curation, investigation, project administration, resources, supervision, visualization. **Maisara Fathi Ahmed Salih:** data curation, investigation, project administration, supervision, validation, visualization. **Heyam Abdelazim Abdelrahim Ali:** data curation, investigation, resources, validation, visualization. **Rame Taha Zeiada Taha:** conceptualization, data curation, investigation, validation, visualization, writing – review and editing. **Loai Osman Elrofaie Osman:** data curation, resources, software, validation, visualization, writing – review and editing.

## Funding

The authors have nothing to report.

## Ethics Statement

Written Informed consent was obtained from the patient herself for publication.

## Conflicts of Interest

The authors declare no conflicts of interest.

## Data Availability

All data generated or analyzed during this study are included in this published article.
